# Thermosensitive heparin‐poloxamer hydrogel encapsulated bFGF and NGF to treat spinal cord injury

**DOI:** 10.1111/jcmm.15478

**Published:** 2020-06-08

**Authors:** Xiaoli Hu, Rui Li, Yanqing Wu, Yi Li, Xingfeng Zhong, Guanyinsheng Zhang, Yanmin Kang, Shuhua Liu, Ling Xie, Junming Ye, Jian Xiao

**Affiliations:** ^1^ Department of Anesthesia The First Affiliated Hospital Gannan Medical University Ganzhou China; ^2^ Molecular Pharmacology Research Center School of Pharmaceutical Science Wenzhou Medical University Wenzhou China; ^3^ School of Chemistry Sun Yat‐sen University Guangzhou China; ^4^ The Institute of Life Sciences Engineering Laboratory of Zhejiang Province for Pharmaceutical Development of Growth Factors Biomedical Collaborative Innovation Center of Wenzhou Wenzhou University Wenzhou China; ^5^ Department of Anesthesia Affiliated Hospital of Guizhou Medical University Guiyang China

**Keywords:** basic fibroblast growth factor (bFGF), heparin‐poloxamer (HP) hydrogel, nerve growth factor (NGF), neuroprotection, spinal cord injury

## Abstract

The application of growth factors (GFs) for treating chronic spinal cord injury (SCI) has been shown to promote axonal regeneration and functional recovery. However, direct administration of GFs is limited by their rapid degradation and dilution at the injured sites. Moreover, SCI recovery is a multifactorial process that requires multiple GFs to participate in tissue regeneration. Based on these facts, controlled delivery of multiple growth factors (GFs) to lesion areas is becoming an attractive strategy for repairing SCI. Presently, we developed a GFs‐based delivery system (called GFs‐HP) that consisted of basic fibroblast growth factor (bFGF), nerve growth factor (NGF) and heparin‐poloxamer (HP) hydrogel through self‐assembly mode. This GFs‐HP was a kind of thermosensitive hydrogel that was suitable for orthotopic administration in vivo. Meanwhile, a 3D porous structure of this hydrogel is commonly used to load large amounts of GFs. After single injection of GFs‐HP into the lesioned spinal cord, the sustained release of NGF and bFGF from HP could significantly improve neuronal survival, axon regeneration, reactive astrogliosis suppression and locomotor recovery, when compared with the treatment of free GFs or HP. Moreover, we also revealed that these neuroprotective and neuroregenerative effects of GFs‐HP were likely through activating the phosphatidylinositol 3 kinase and protein kinase B (PI3K/Akt) and mitogen‐activated protein kinase/extracellular signal‐regulated kinase (MAPK/ERK) signalling pathways. Overall, our work will provide an effective therapeutic strategy for SCI repair.

## 1 INTRODUCTION

Spinal cord injury (SCI) is one of the most serious traumatic diseases that affects half a million people each year, especially for young adults. After suffering from contusion, compression or traumatic accidents, the epicentre region of spinal cord undergoes a complex pathological change, including primary injury and secondary injury.^1^, ^2^ The former directly results in tissue damage and neural cell death. The latter cascade reaction frequently generates the formation of free radical and triggers chronic inflammatory response, leading to cystic cavities and scar tissue forms.^3^ These two pathological progressions of SCI greatly intensify the failure of axonal regeneration, which seriously affects a patient's quality of life and life expectancy. Unfortunately, up to now, researchers have not created an available approach for effectively stimulating and guiding axonal growth to ensure functional recovery after SCI.

Over the last decades, numerous strategies have been attempted to locally apply growth factors (GFs) to improve morphological and behavioural outcomes after SCI. Moreover, these GFs have been demonstrated to modulate the survival of damaged neurons, facilitate axonal sprouting and regeneration, as well as block inhibitory molecules.^4^, ^5^ As two typical GFs, basic fibroblast growth factor (bFGF) and nerve growth factor (NGF) are capable of stimulating neurogenesis, axonal growth and angiogenesis in the central nervous system.^6^, ^7^, ^8^, ^9^ Treatment of exogenous bFGF shifted astroglial population towards neurogenic radial/progenitor bipolar glia cells, which contributed to decrease astrocyte reactivity and glial scar formation at later stage of SCI.^7^ NGF‐loaded gelatin nanostructured lipid carriers showed significant therapeutic effects on improving locomotion recovery of SCI through inhibiting endoplasmic reticulum (ER) stress‐induced apoptosis.^10^ However, during SCI, some GFs expression, such as bFGF and NGF, are greatly reduced within the lesion site.^11^ These declining GFs production has been shown to cause protracted neuronal loss and neurological dysfunction. Thus, an optimal spinal cord regeneration need supplement of exogenous GFs. Considering administration of exogenous GFs exists some drawbacks, such as their short half‐life and poor penetrability of the blood spinal cord barrier (BSCB).^12^ Therefore, it is necessary for developing a drug delivery strategy that can effectively deliver bFGF and NGF into the spinal cord injury site to enhance neuronal development and axonal regeneration.^13^, ^14^

Hydrogels are networks of polymer chains that possess excellent biocompatibility, biodegradability, permeability and biomechanical properties. Several studies have confirmed that these water‐swollen cross‐linked hydrogels are suitable for injecting into the cystic cavity to re‐establish a favourable microenvironment for facilitating the repair and reconstruction of damaged or defected tissues.^15^, ^16^, ^17^ In order to effectively improve growth factor (GF) therapy on SCI, we developed a novel thermosensitive heparin‐poloxamer (HP) hydrogel. This thermosensitive hydrogel has several advantages^18^: (a) a high affinity for various GFs; (b) controlling these GFs release in a steady fashion; (c) protecting them from enzymolysis, and (d) avoiding side effects of high GF concentrations at the injectable site. Thus, after incorporating bFGF and NGF into HP vehicle, this GFs‐HP system not only reduces the frequency of administration, but also sustains NGF and bFGF release for a sufficient period of time to persistently treat SCI.

It is well known that different GFs have different biological effects via interacting their corresponding receptors. NGF usually bind two receptors, namely high‐affinity receptor tyrosine kinase A (TrkA) and low‐affinity receptor p75^NTR^. TrkA possesses tyrosine protease activity to trigger the activation of diverse downstream signalling mechanisms, including MAPK, phosphatidylinositol 3 kinase and protein kinase B (PI3K/Akt) and PLCγ pathways.^19^ The p75^NTR^ receptor is a transmembrane glycoprotein that generally activates the NF‐κB signalling to exert distinct roles on regulating neuronal survival, development and maturation.^20^ The specific role of NGF in repairing SCI depends on which receptor to bind. Perhaps an exact technique is to examine the expression of NGF and its receptors in different tissues. bFGF is the widely identified GF that can induce angiogenesis and neurogenesis through two known receptors, the high‐affinity fibroblast growth factor FGF receptor (FGFR) and the low‐affinity heparan sulphate proteoglycan (HSPG), respectively. Among them, FGFR1 was studied frequently and elicited the activation of Ras‐Raf‐MAPK, JNK and PI3K/Akt signalling cascades to promote cellular survival, growth and proliferation via forming bFGF‐FGFR1 complex.^21^ However, whether these two GFs in this study act on their receptors alone or together to activate the PI3K/Akt and/or MAPK/ERK pathways remains to be further studied.

In the present study, we hypothesized that the sustained release of bFGF and NGF from GFs‐HP could interact with cell membrane receptors, including FGFR1 and Trk A, to activate the downstream transduction pathways of PI3K/Akt and mitogen‐activated protein kinase/extracellular signal‐regulated kinase (MAPK/ERK). Activation of these two signalling cascades was able to further increase the survival of neurons, upregulate intrinsic protein expression and enhance axon regrowth, as well as improve the functional recovery after acute SCI in rats. We firstly observed the micromorphology of HP containing with/without GFs using scanning electron microscopy (SEM), In addition, the repair and regeneration of injured spinal cord by in situ injection of GFs‐HP hydrogel was comprehensively evaluated through immunohistochemistry, immunofluorescence staining and Western blotting. This approach may provide GFs‐HP as a potential agent for repairing acute SCI.

## 2 MATERIALS AND METHODS

### 2.1 Preparation of HP and GFs‐HP hydrogels

Based on the previous description, heparin‐poloxamer (HP) was prepared according to the EDC/NHS method.^22^ Briefly, poloxamer 407 (Badische Anilin Soda Fabrik Ga) reacted with 1.3 mmol/L 4‐nitrophenyl chloroformate and diamino ethylene to obtain a mono amine‐terminated Poloxamer (MATP). Then, this intermediate was coupled with heparin salt by EDC and NHS in 2‐(N‐morpholine) sulphonic acid (MES) buffer for 1 day at 25°C. The reactive mixture was dialysed for 3 days and lyophilized to obtain HP powder. GFs‐HP hydrogel, consisted of HP hydrogel, bFGF and NGF, was prepared using the cold method. Briefly, HP powders were dissolved in fresh saline and gentle stirred at 4°C for a period of 24 hours to obtain a clear solution. Then, bFGF and NGF organic solution (2.00 and 1.42 mg/mL, respectively, obtained from Key Laboratory of Biotechnology and Pharmaceutical Engineering, Wenzhou Medical University) were immediately added to the HP solution under vigorous magnetic agitation to obtain their final concentration with 17% (w/w), 1 and 1 mg/mL, respectively. The final mixture was stored at 4°C for subsequent experiments. The whole process was carried out under aseptic conditions.

### 2.2 Micromorphology of HP and GFs‐HP hydrogels

The micromorphology of the dehydrated HP and GFs‐HP hydrogels was characterized using scanning electron microscopy (SEM, Hitachi H‐7500). Briefly, after freezing with l liquid nitrogen, the frozen HP hydrogels were dried in a freeze dryer for 24 hours. Subsequently, they were carefully crosscut and placed into the conductive glue for gold‐plating. Lastly, HP morphology was observed by SEM.

### 2.3 SCI model and orthotopic injection

Adult female Sprague Dawley (SD) rats (220‐250 g) were purchased from the Laboratory Animal Center. The animal use and care protocol were conformed to the Guide for the Chinese National Institutes of Health (NIH) and approved by the Animal Care and Use Committee of Wenzhou Medical University. Before the production model of SCI, animals were maintained for at least 7 days to adjust to the standardized laboratory temperature (23 ± 2°C), humidity (35%‐60%) and a light‐dark cycle (12:12 hours). The surgical procedures to construct the impact SCI model were performed as previously described.^23^ In short, after anesthetizing by 10% (w/v) chloral hydrate (3.5 mL/kg, i.p.), the animals were exposed T9‐T10 lamina using rongeur. Then, the 2.5‐mm stainless steel impactor tip was positioned over the midpoint of T9‐T10 and dropped from a height of 12.5 mm (150 kdyn force) to strike the exposed spinal cord. Afterwards, dissected muscle, fascial and skin were sutured layer by layer with 4‐0 absorbable lines. The sham group only exposed spinal cord without SCI.

Following SCI, the injured rats were further divided into 4 groups (n = 10 for each): SCI, HP hydrogel, free GFs and GFs‐HP hydrogel. Each animal was orthotopic injection of HP, free GFs or GFs‐HP solution with the volume of 20 µL using microsyringe. The rats were administered with penicillin for consecutive 7 days. The bladder was massaged triple daily until the bladder function returned to normal.

### 2.4 Locomotion recovery evaluation

Basso‐Beattie‐Bresnahan (BBB) score and footprint analysis were conducted to evaluate the restoration of hindlimb locomotor function. In brief, at predetermined time points (0, 3, 7, 14, 21 and 28 days), the movement of rats’ hind limbs were recorded by trained investigators who were blind to the experimental conditions. The BBB score ranged from 0 to 21 points, where 0 points indicated complete paralysis and 21 points represented normal locomotion.

At the time point of 28 day post‐surgery, a footprint analysis was performed by dipping the animal's hindpaws in dye. All of the rats are tested in a confined walkway (an 8.2 cm wide by 42‐cm‐long white paper) with a dark shelter at the end. Each experiment/group was randomly selected at least 5 rats to evaluate motor function recovery.

### 2.5 Preparation of spinal cord tissue

Rats in each group were sacrificed at 28 day after SCI. The animals were anesthetized with 10% chloral hydrate (3.5 mL/kg, i.p.). Then, the hearts were perfused with normal saline. After the saline infusion, the spinal segment of the injury centre (0.5 cm length) was harvested and stored at −80°C immediately for immunoblotting. For other experiments, such as haematoxylin and eosin (HE) staining, Nissl staining, immunohistochemistry and immunofluorescence, the prepared process was described previously.^24^ Briefly, after washing away the blood, the epicentre sections of injured spinal cord (0.5 cm) were extracted and fixed in 4% paraformaldehyde overnight. Next, the samples were sequentially underwent rinsing, dehydration, permeabilization and embedding. Lastly, longitudinal or transverse sections were then cut into 5 µm on a cryostat (Leica Microsystems Wetzlar GmbH).

### 2.6 Histological analysis

The prepared transverse sections were implemented HE Staining and Nissl following the manufacturer's instructions. It should be emphasized that the sections were dyed with haematoxylin for 5 minutes, with eosin for 2 minutes and with cresyl violet for 8 minutes in our experimental condition. Then, the images were captured using an optical microscope (Nikon ECLIPSE Ti‐S, Ruikezhongyi Company).

### 2.7 Immunofluorescence and immunohistochemistry labelling

After dewaxing and hydration, the transverse or longitudinal paraffin sections were incubated in 3% H_2_O_2_ for 15 minutes and then in blocking solution for 1 hour at room temperature. Subsequently, the sections were incubated at 4°C over‐night with the following primary antibodies: NeuN (ab‐177487, Abcam, 1:500), GFAP (ab7260, Abcam, 1:1000) and NF‐200 (ab‐8135, Abcam, 1:1000). The next day, redundant antibodies on the tissue sections were washed with PBST for 15 minutes. The following procedures for immunofluorescence and immunohistochemistry were different. For the former, the sections were incubated with Alexa Fluor 488 donkey anti‐rabbit secondary antibody (ab‐150073, Abcam, 1:1000) at 37°C for 1 hour. After incubating with DAPI for 7 minutes, the samples were imaged using a confocal fluorescence microscope (Nikon). For immunohistochemistry, followed by incubated with horseradish peroxidase‐conjugated secondary antibodies at 37°C for 1 hour, the sections were stopped with 3, 3‐diaminobenzidine (DAB) and stained with haematoxylin. The images were captured using a confocal fluorescence microscope (Nikon, Japan). The quantification of each density was performed using the ImageJ software.

### 2.8 Western blotting

Total protein from the spinal cord tissue was purified using protein extraction reagents containing 1% protease and phosphatase inhibitors. The protein concentration of each sample was quantified with Carmassi Bradford reagents (Thermo). An equivalent amount of protein (60 µg) was separated by 10% SDS‐PAGE and transferred onto PVDF membranes (Bio‐Rad). After blocking with 5% (w/v) non‐fat milk, the membranes were further incubated with primary antibody solutions overnight at 4°C. The following primary antibodies were including: TrkA (ab‐76291, Abcam, 1:5000), FGFR1 (ab‐58516, Abcam, 1:1000), *P*‐AKT (sc‐514032, Santa Cruz, 1:1000), AKT (sc‐81434, Santa Cruz, 1:1000), *P*‐ERK (sc‐16982, Santa Cruz, 1:1000), ERK (sc‐514302, Santa Cruz, 1:1000), Bcl‐2 (60178‐1‐Ig, proteintech, 1:3000), Bax (60267‐1‐Ig, proteintech, 1:2000), cleaved caspase‐3 (sc‐373730, Santa Cruz, 1:500), GAP43 (ab75810, Abcam, 1:10 000), GFAP (ab7260, Abcam, 1:2000), NF‐200 (ab8135, Abcam, 1:5000) and GAPDH (K200057M, Solarbio, 1:5000). After three washed with TBST, the membranes were incubated with a 1:10 000 dilution of horseradish peroxidase‐conjugated secondary antibodies for 60 minutes at room temperature. Finally, signals were visualized by Chemi DocXRS + Imaging System (Bio‐Rad). All experiments were repeated three times.

### 2.9 Statistical analysis

All data were presented as the mean ± standard error of the mean (SEM). For unpairwise comparisons, the Student's *t* test was used. For multiple comparisons, the one‐way analysis of variance (ANOVA) with Bonferroni's multiple comparisons for post hoc analysis was used. All statistical analyses were performed with statistical software GraphPad Prism software Version 5 (GraphPad Software, Inc), and *P* values < .05 were considered statistically significance.

## 3 RESULTS

### 3.1 HP hydrogel loaded with GFs retain the 3D porous structure and thermal‐sensitive characteristic

**FIGURE 1:**
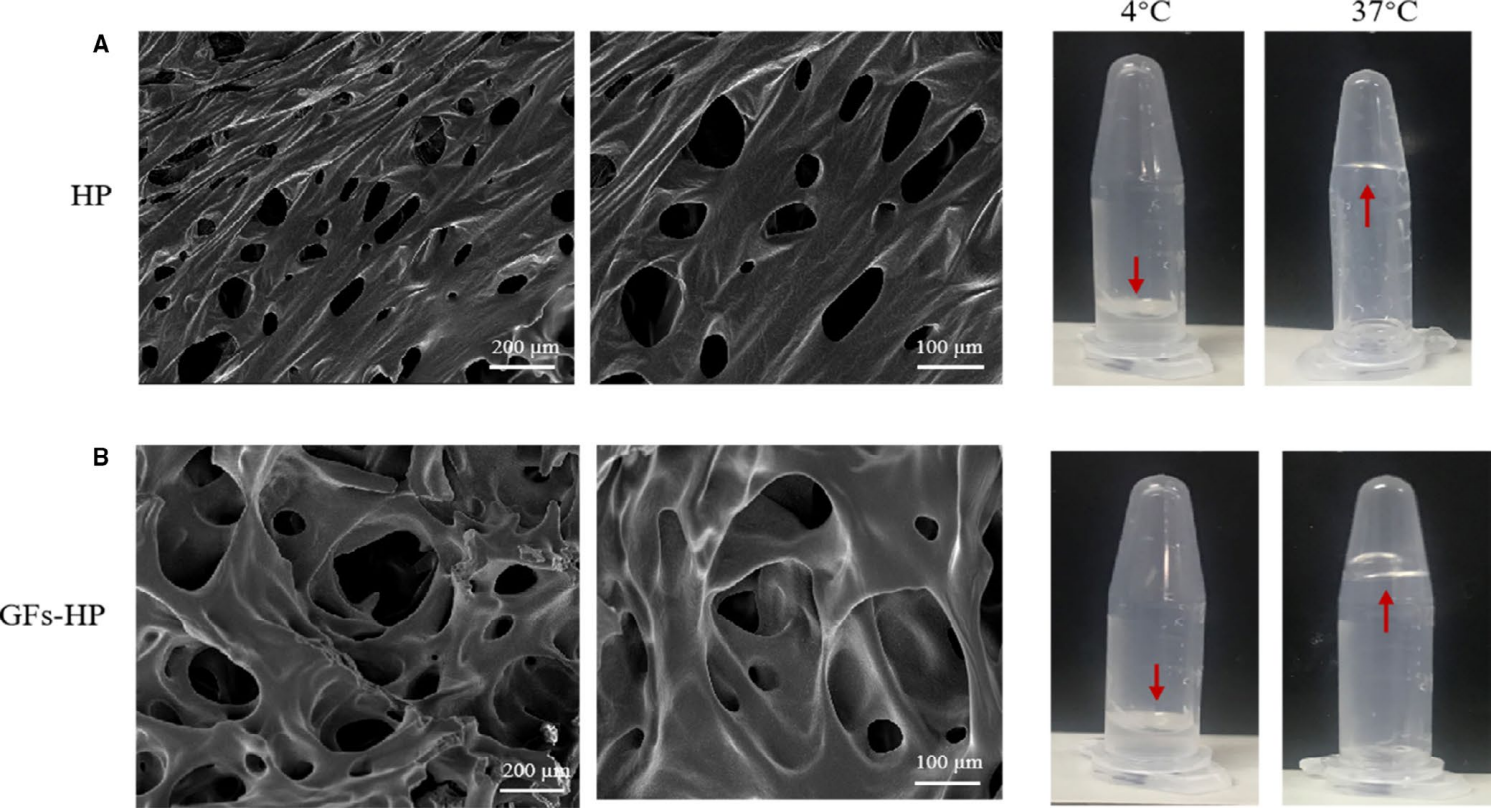
Characterization of HP and GFs‐HP hydrogel. A, SEM images and the thermosensitive property of HP hydrogel. B, SEM images and the thermosensitive property of GFs‐HP hydrogels. The SEM images of both hydrogels were photographed at low magnification (left, scale bar: 200 µm) and high magnification (right, scale bar: 100 µm), respectively. Meanwhile, the temperature‐sensitive characteristic of both hydrogels was tested at 4 and 37°C condition

Previously, we confirmed that the HP solution with 17% (w/w) concentration had a controlled phase alteration temperature.^25^ Thus, this concentration of HP was also be adopted to investigate the microstructure and gelation properties. According to the SEM observation, HP presented a sponge‐like 3D porous structure that interconnected into a mesh shape (Figure 1A). Moreover, this liquid HP solution at 4°C could quickly transitioned to the hydrogel (gel) state upon heating to a body temperature of 37°C (Figure 1B). Similarly, the 3D porous structure and thermosensitive characteristic were still retained after adding bFGF and NGF to the HP (Figure 1A and B). Collectively, this 3D network structure of HP is suitable for loading various GFs and the temperature sensitivity of GFs‐HP is beneficial for orthotopic administration.

### 3.2 GFs‐HP promotes the motor functional recovery after SCI

**FIGURE 2:**
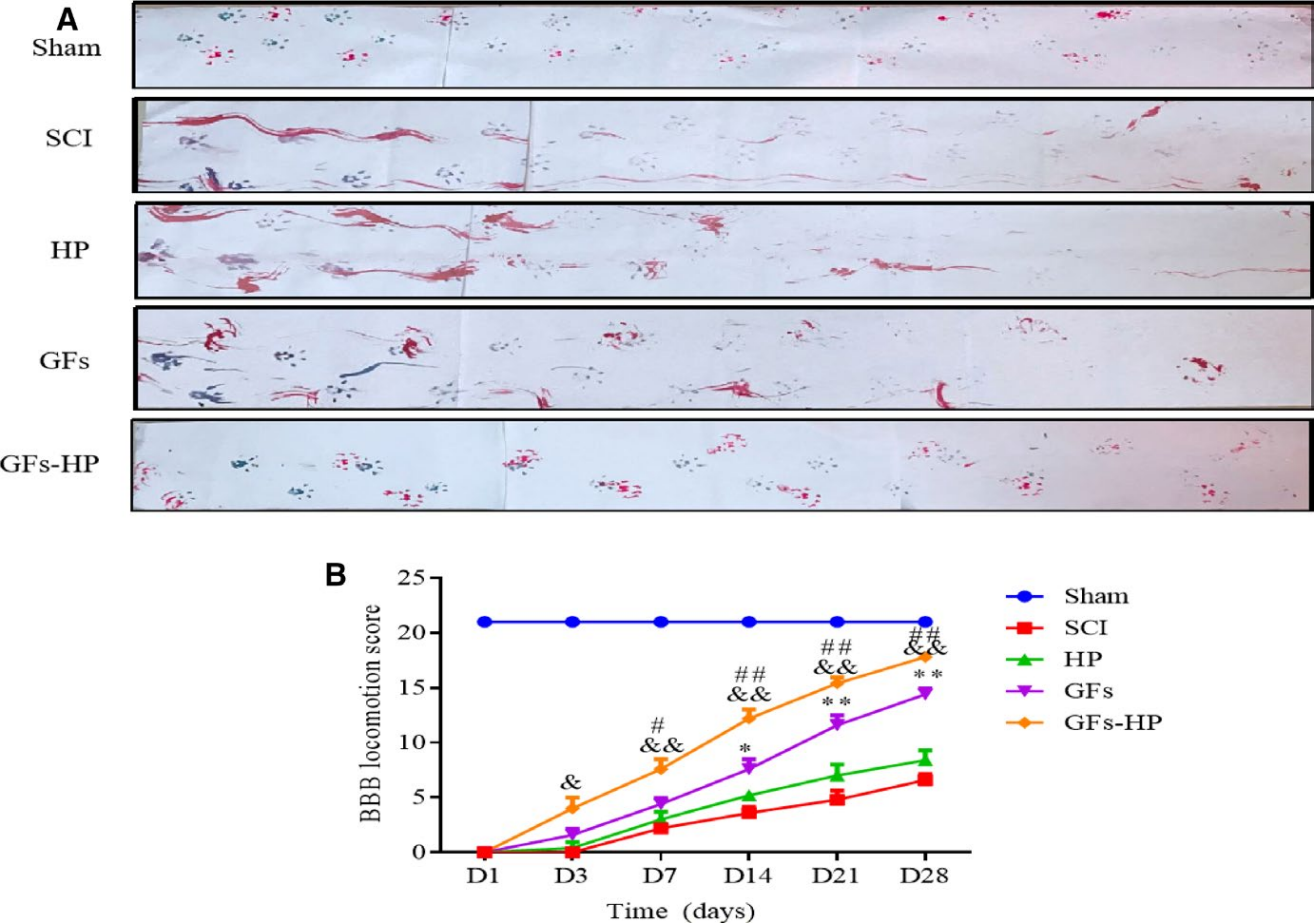
GFs‐HP improves the motor function recovery in SCI model rats. A, Footprint analyses of the sham, SCI, HP, GFs and GFs‐HP groups at 28 d post‐injury. B, BBB locomotion assessments of different groups at 1, 3, 7, 14, 21, 28 d post‐SCI. Values were expressed as the mean ± SEM, n = 10 per group. ^&^ *P* < .05, ^&&^ *P* < .01 vs the SCI group.^*^ *P* < .05, ^**^ *P* < .01 vs the SCI group. ^#^ *P* < .05, ^##^ *P* < .01 vs the GFs group

To evaluate whether in situ GFs‐hydrogel injection therapy could effectively promote the recovery of motor function, footprint analysis and BBB locomotion scores were performed according to the method reported previously.^18^ As shown in Figure 2A, the footprint test could intuitively reflect the restoration of hind leg movement in each group at 28 days post‐injury (dpi). In the SCI and HP groups, injured rats still dragged their hind legs, leaving unambiguous footprint. In contrast, in the GFs and GFs‐HP groups, the footprint recordings exhibited coordinated crawling with very little toe dragging. Moreover, compared to the GFs group, GFs‐HP group exhibited better coordination. BBB evaluation showed that the hindlimb motion was lost immediately after SCI and subsequently manifested modest time‐dependent recovery. Surprisingly, BBB score between the GFs‐HP group and SCI group exhibited a statistical difference as early as 3 days post‐surgery. At subsequent time (7‐28 days), the BBB scores in the GFs‐HP group were markedly higher than that in the GFs solution group, which almost reached to sham group at day 28 (Figure 2B). All these data indicate that the GFs‐HP hydrogel (OI) group showed the best motor functional recovery compared with other treated groups, which is suitable for SCI repair.

### 3.3 GFs‐HP improves morphologic degeneration in SCI rats

**FIGURE 3:**
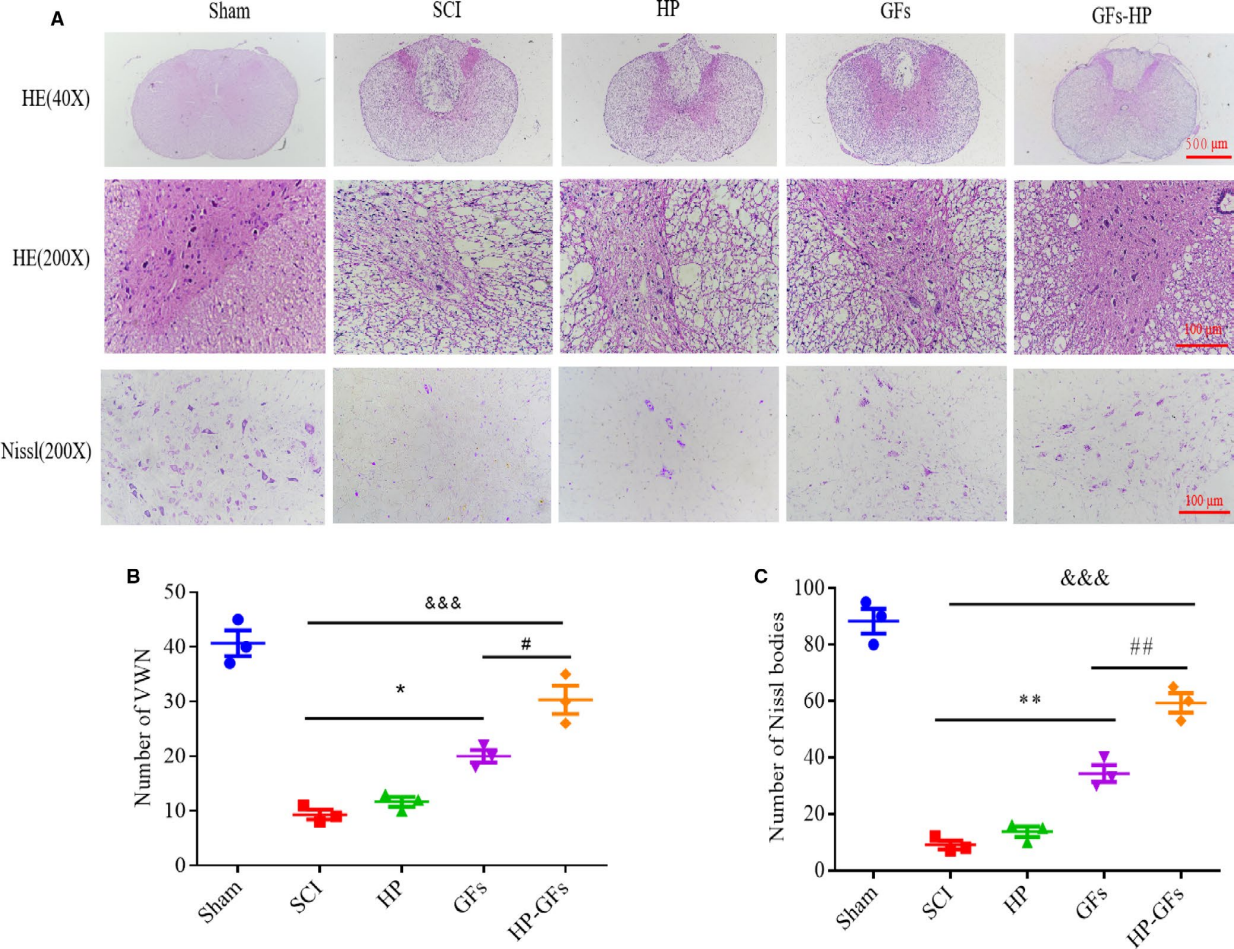
GFs‐HP decreases the damage of tissue structure and the loss of neurons at 28 d after SCI. A, Representative images from HE staining (40× and 200×) and Nissl staining (200×) on transverse sections in sham, SCI, HP, GFs and GFs‐HP groups at 28 dpi. Scale bar: 500 µm for low magnification images and 100 µm for high magnification ones. B, Counting analysis of the number of VMNs from (A) of HE staining. C, Quantitative analysis of the Nissl bodies numbers from Nissl staining. Values were expressed as the mean ± SEM, n = 4 per group. ^&&&^ *P* < .001 vs the SCI group. ^*^ *P* < .05, ^**^ *P* < .01 vs the SCI group. ^#^ *P* < .05, ^##^ *P* < .01 vs the GFs group

In order to verify GFs‐HP hydrogel could improve tissue damage and reduce neuronal loss, HE and Nissl staining in each group were detected at 28 days after injury, As shown in Figure 3A, sham group showed integral structure, manifesting in the clear grey and white outline and intensive ventral motor neurons (VMNs) filling in the grey matter. However, the dorsal white matter in SCI and HP groups was severely damaged and formed a huge cavity area, accompanying by scale VMNs survival. Compared with SCI and HP groups, GFs treatment showed large improvement of tissue morphology with less neuronal necrosis and more VMNs existence. Furthermore, the GFs‐HP group almost achieved the healing degree to the sham group. Nissl staining also exhibited a similar trend with HE staining. Specifically, there were a large number of Nissl bodies existed in the sham group, while only few Nissl bodies were observed in SCI and HP groups. Treatment of GFs and GFs‐HP, especially for the latter drug, significantly increases the numbers of Nissl bodies (Figure 3A). The ranking of the numbers of VMNs and Nissl bodies from high to low was as follows: sham group > GFs‐HP hydrogel group > GFs group > HP hydrogel group > SCI group (Figure 3B and C). All of these findings indicate that GFs‐HP hydrogel can exert a best therapeutic effect on protecting neuronal survival and ameliorating the pathological morphology in the injured spinal cord.

### 3.4 GFs‐HP hydrogel enhances neuronal survival and suppresses its apoptosis in vivo

**FIGURE 4:**
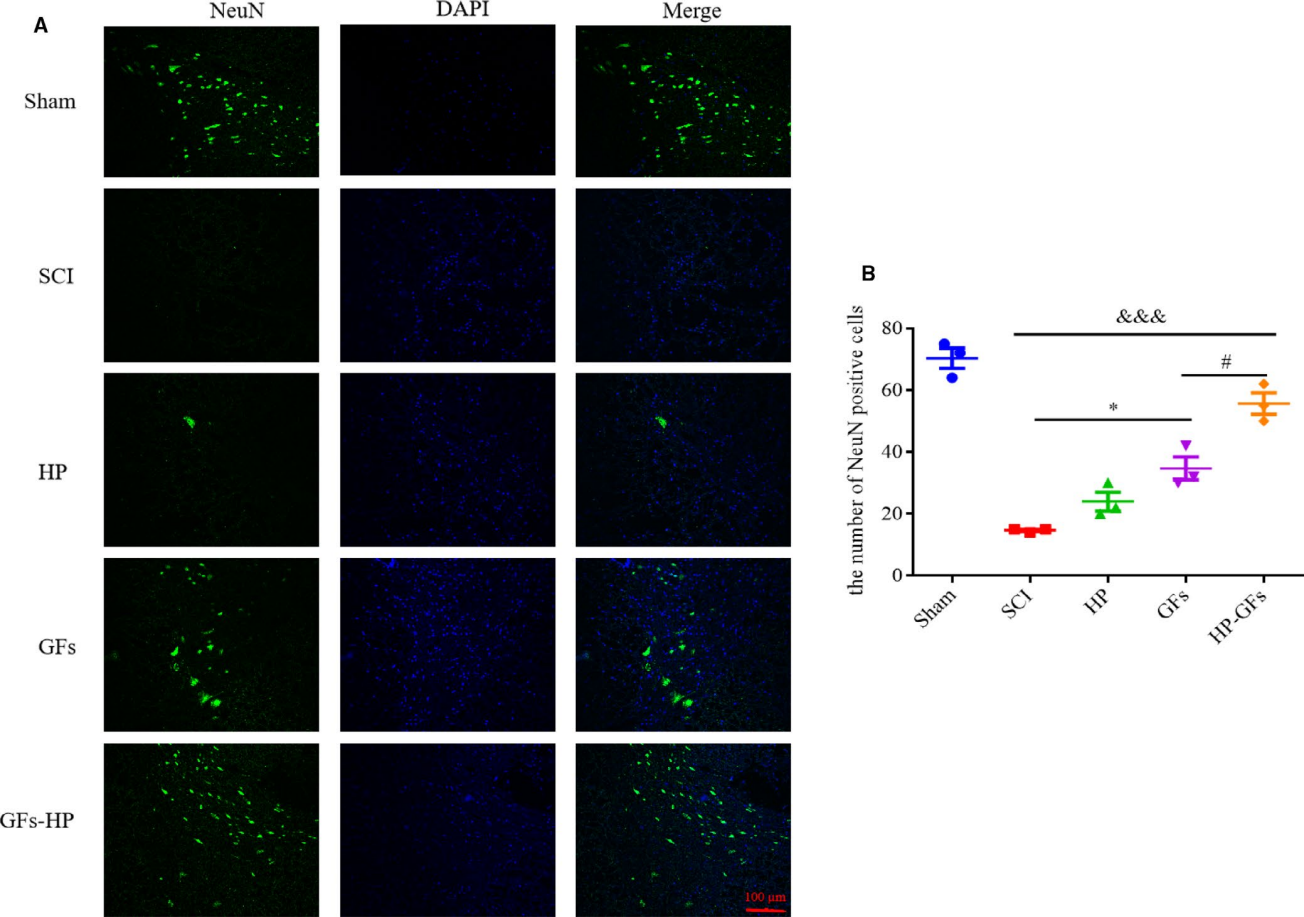
GFs‐HP hydrogel enhances neuronal survival at 28 d after SCI. A, Fluorescence images of transverse sections show the expressions of NeuN in the sham, SCI, HP, GFs and GFs‐HP groups. Scale bar = 100 µm. B, Quantifying the number of NeuN^+^‐positive cells from (A). Results were expressed as the mean ± SEM, n = 4 per group. ^&&&^ *P* < .001 vs the SCI group, ^*^ *P* < .05 vs the SCI group, ^#^ *P* < .05 vs the GFs group

Traumatic SCI often initiates neurological dysfunction, apoptosis and even necrosis, which severely impedes nerve regeneration and function recovery.^26^ To further evaluate the effects of GFs‐HP on promoting neuronal survival and reducing its apoptosis after SCI, immunofluorescence staining for NeuN and Western blotting for Bcl‐2, BAX and cleaved caspase‐3 was performed in the tested five groups at 28 dpi. As shown in Figure 4A and B, the number of NeuN‐positive cells in the SCI and HP groups was few, whereas the GFs treatment significantly increased the number of NeuN‐positive cells, but this trend was inferior to the GFs‐HP hydrogel (GFs‐HP group vs GFs group: *P* < .05).

**FIGURE 5:**
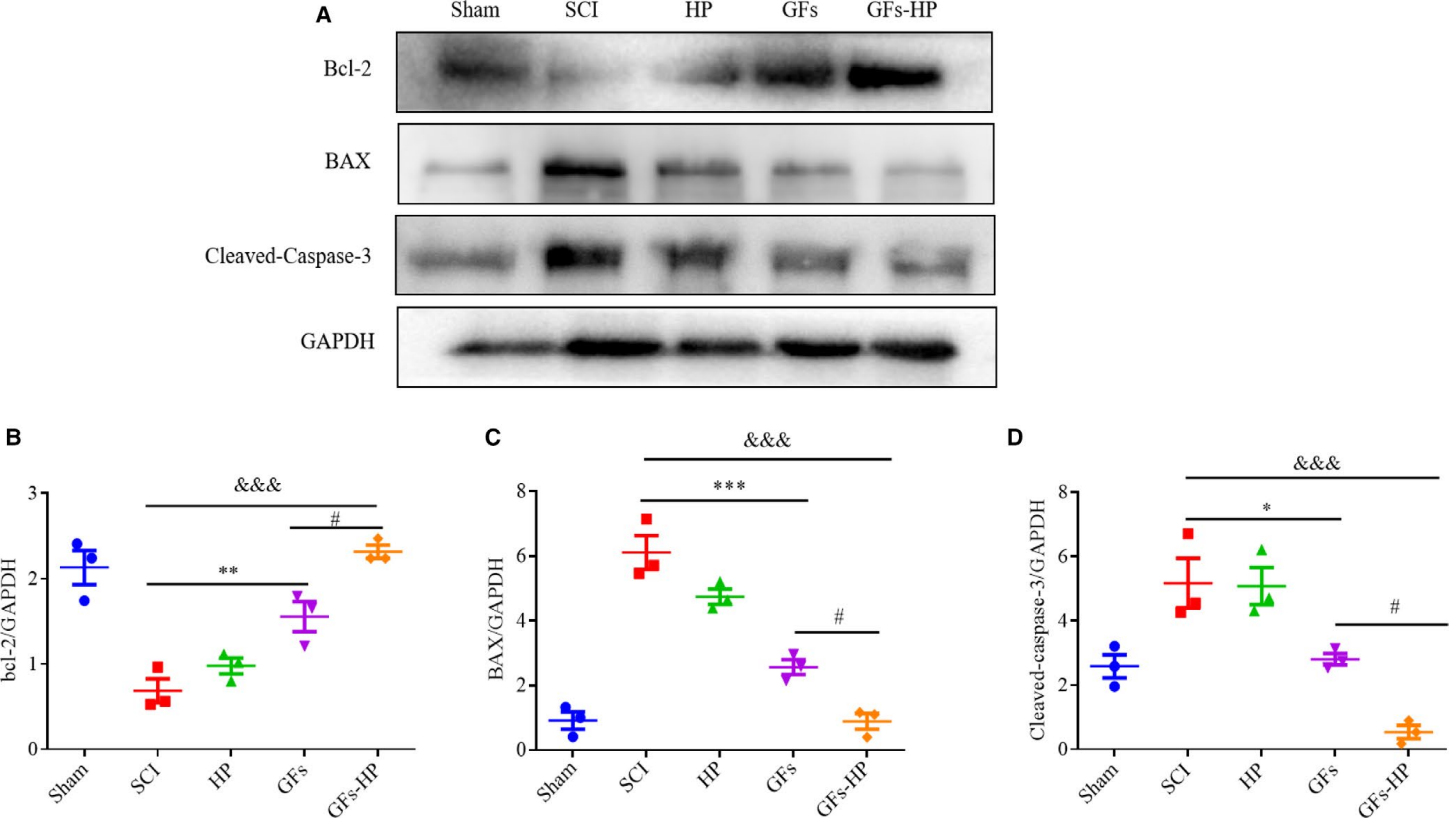
GFs‐HP hydrogel reduces neuronal apoptosis at 28 d after SCI. A, Representative immunoblotting bands of Bcl‐2, BAX, cleaved caspase‐3 in the sham, SCI, HP, GFs and GFs‐HP groups. GAPDH was used as was used as the loading control and for band density normalization. B‐D, The optical density analysis of Bcl‐2, BAX, cleaved caspase‐3 proteins in five groups. Values were expressed as the mean ± SEM, n = 4 per group. ^&&&^ *P* < .001 vs the SCI group. ^*^ *P* < .05, ^**^ *P* < .01, ^***^ *P* < .001 vs the SCI group. ^#^ *P* < .05 vs the GFs group

Next, the expressing level of typical apoptotic proteins (BAX and cleaved caspase 3) and anti‐apoptotic protein (Bcl‐2) was further tested by Western blotting (Figure 5A). The results revealed that, compared to the sham group, the expression of apoptotic proteins was significantly upregulated but anti‐apoptotic protein was presented more lower in SCI and HP groups. GFs‐HP substantially reversed this trend, and this inhibiting degree was better than GFs treatment (Figure 5B‐D, GFs‐HP group vs GFs group: all *P* < .05). All of the above data indicate that HP hydrogel combination of NGF and bFGF plays a significant beneficial role in promoting neuronal survival and reducing their apoptosis after SCI.

### 3.5 GFs‐HP hydrogel promotes axonal rehabilitation and plasticity

**FIGURE 6:**
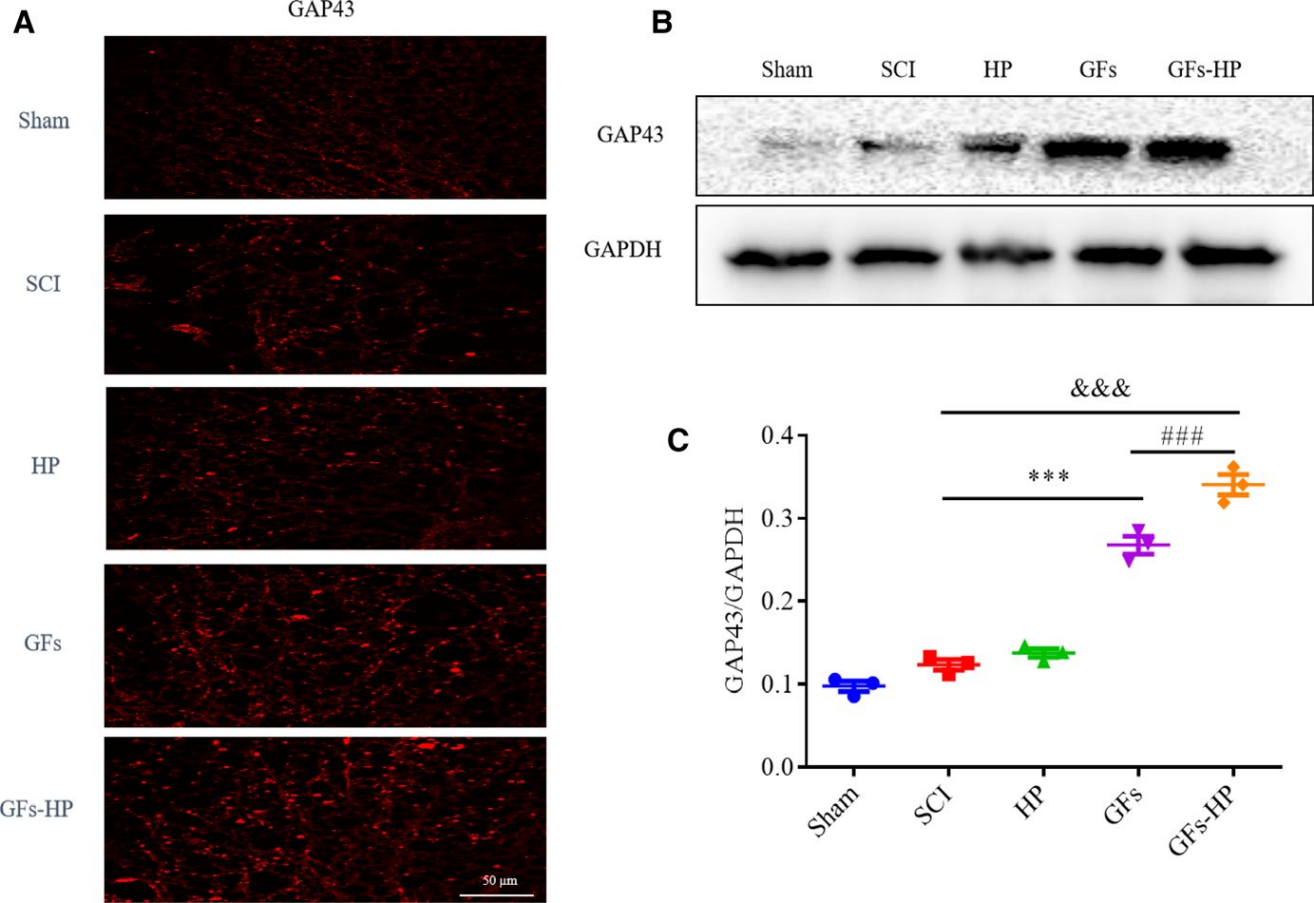
GFs‐HP hydrogel promotes axonal growth at 28 d after SCI. A, Representative fluorescence images for GAP43 of transverse sections from the injured spinal cord in each group. Scale bar = 50 µm. B, Protein expressions of GAP43 in each group via Western blotting detection. C, Densitometric analyses of GAP43 from (B). GAPDH was used for band density normalization. Values were expressed as the mean ± SEM, n = 4 per group. ^&&&^ *P* < .001 vs the SCI group. ^***^ *P* < .001 vs the SCI group. ^###^ *P* < .001 vs the GFs group

The growth‐associated protein 43 (GAP43) is an axon growth‐related protein that regulates neurite growth, synaptic development and nerve cell regeneration.^27^ Upon maturation of the spinal cord, GAP43 is downregulated by mostly neurons. After nerve damage, the regenerative neurons can secrete a certain amount of GAP43 to mediate axonal extension and plasticity.^28^ Thereby, the detection of GAP43 expression can indirectly reflects the circumstances of axon outgrowth and synapse formation.^29^ In this study, we tested GAP43 expression in each group using Western blotting and immunofluorescence staining. As shown in Figure 6A, compared to the SCI and HP groups, the GAP43^+^ signals were largely enhanced in GFs and GFs‐HP groups. Western blotting also showed that the level of GAP43 expression was pretty low in the sham, while in the SCI and HP groups, this protein level was evidently increased. Moreover, this trend of GAP43 expression was further increased in GFs and GFs‐HP groups, especially for the latter group (Figure 6B and C, GFs‐HP group vs GFs group: *P* < .001). These results proved that co‐delivery of bFGF and NGF with HP was favourable for axon outgrowth and plasticity.

### 3.6 GFs‐HP hydrogel enhances axonal regeneration and attenuates the reactive astrogliosis

**FIGURE 7:**
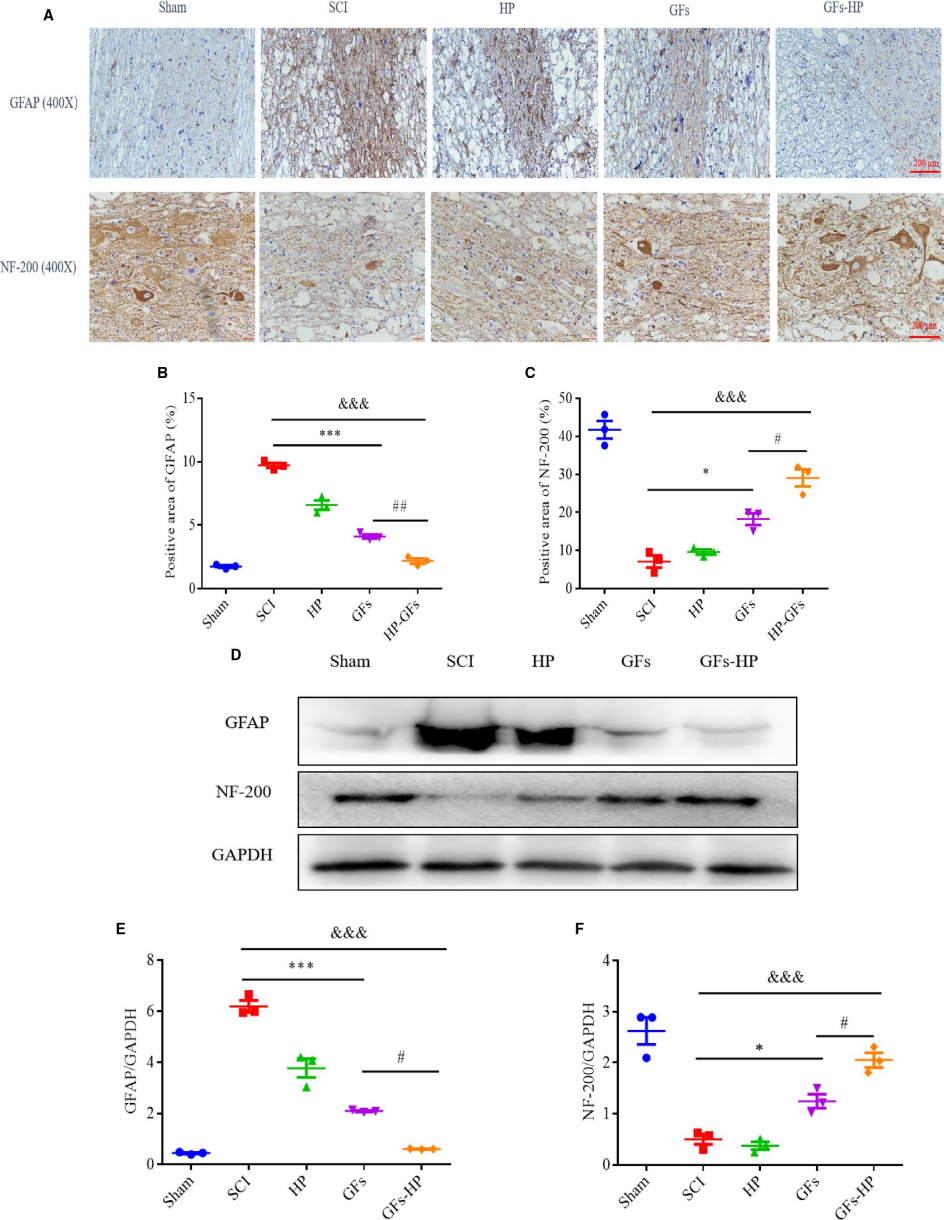
GFs‐HP hydrogel promotes axonal regeneration and attenuates the reactive astrogliosis at 28 d after SCI. A, Immunohistochemical staining of NF‐200 and GFAP of longitudinal sections in the sham, SCI, HP, GFs and GFs‐HP groups at 28 d after SCI. Scale bar = 50 µm. B‐C, Quantitative analysis of GFAP and NF‐200‐positive area from A. D, Western blotting detected the protein levels of GFAP and NF‐200 in each group, GAPDH served as a protein loading control. E‐F, Quantification of GFAP and NF‐200 from D. Values were expressed as the mean ± SEM, n = 4 per group. ^&&&^ *P* < .001 vs the SCI group. ^*^ *P* < .05, ^***^ *P* < .001 vs the SCI group. ^#^ *P* < .05, ^##^ *P* < .01 vs the GFs group

SCI leads to astrocyte's activation to form a glial scar around the injury site, which severely hinders neural regeneration and axonal regrowth.^30^ To investigate whether GFs‐HP could reduce glial scar formation to guide axon growth, the spinal cord sections in all experimental animals were stained for glial fibrillary acidic protein (GFAP, a marker of astrocyte activation) and neurofilament‐200 (NF‐200, marking for axon). Immunohistochemistry result showed that GFAP was presented abundance and dense at 28 dpi in the SCI and HP groups. In contrast, in the other two treating groups, especially for the GFs‐HP group, the GFAP staining showed an tremendous decrease, which was nearly closed to the sham group (Figure 7A and B), whereas NF‐200 staining intensity was exhibited an opposite tendency; that is, the ranking of NF‐200‐positive areas was as follows: sham group > GFs‐HP hydrogel group > GFs group > HP hydrogel group > SCI group (Figure 7A and C). Similarly, Western blotting detection of GFAP and NF‐200 expression in each group was consistent with the immunohistochemistry analysis (Figure 7D‐F). these results suggest that GFs‐HP hydrogel group has the best therapies for preventing glial scar formation to guide axon growth.

### 3.7 Therapeutic effects of GFs‐HP are linked to stimulate corresponding receptors to activate PI3K/Akt and MAPK/ERK pathways

**FIGURE 8:**
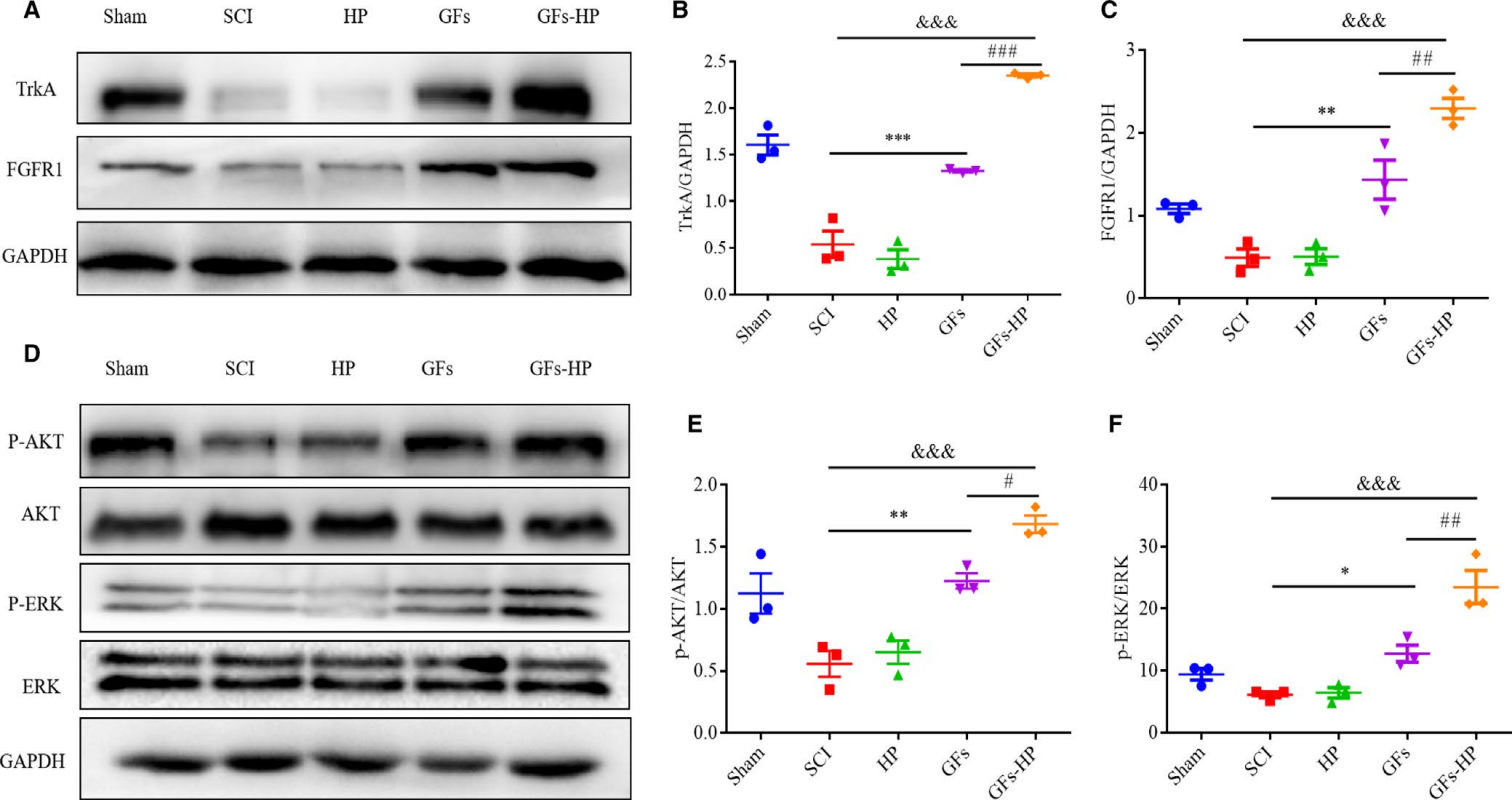
GFs‐HP hydrogel treatment through receptor‐medicated PI3K/AKT and MAPK/ERK activation. A, Immunoblotting for TrkA and FGFR1 in each group. B‐C, The optical density analysis of TrkA and FGFR1 from A. D, Immunoblotting for *P*‐AKT, AKT, *P*‐ERK and ERK in the spinal cord segment at the contusion epicentre. GAPDH was used for band density normalization. E‐F, Quantifying the ratio of p‐AKT/AKT, p‐ERK/ERK from (D). Values were expressed as the mean ± SEM, n = 4 per group. ^&&&^ *P* < .001 vs the SCI group. ^*^ *P* < .05, ^**^ *P* < .01, ^***^ *P* < .001 vs the SCI group, ^#^ *P* < .05, ^##^ *P* < .01, ^###^ *P* < .001 vs the GFs group

Trk A and FGFR1 are high‐affinity receptors for interacting with NGF and bFGF, respectively.^31^ Both NGF/Trk A and bFGF/FGFR1 complexes can activate some downstream signalling cascades, such as PI3K/Akt and MAPK/ERK, to trigger several biological effects including neuronal survival, axonal outgrowth and synaptic plasticity.^32^ To explore whether GFs‐HP ameliorated SCI recovery was related to Trk A and/or FGFR1‐induced PI3K/Akt and MAPK/ERK activation, we first measured the expression levels of Trk A and FGFR1 in each group at 28 dpi by Western blotting. As expected, the expression of both receptors in GFs was higher than that of the SCI and HP groups, while the expression of Trk A and FGFR1 in the GFs‐HP hydrogel group was the highest in all treatment groups (Figure 8A‐C).

To further reveal whether initiated GF‐receptor expression was contributed to activate the downstream signalling pathways of PI3K/Akt and MAPK/ERK, the expression levels of *P*‐AKT, AKT, *P*‐ERK and ERK in five groups at the 28 days post‐surgery were tested by Western blotting. The results showed that the ratio of *P*‐AKT/AKT and *P*‐ERK/ERK in the free GFs and GFs‐HP hydrogel groups exhibited a marked increase, compared with the SCI and HP hydrogel groups. Furthermore, the GFs‐HP hydrogel group showed the highest ration among the five groups (Figure 8D‐F). These data indicate that the underlying mechanism of GFs‐HP hydrogel increasing neurological and functional recovery after SCI may be involve in binding Trk A and FGFR1 receptors to activate the MAPK/ERK and PI3K/Akt signalling pathways.

## 4 DISCUSSION

Our previous studies have utilized HP hydrogel with encapsulated single GF, such as acidic fibroblast growth factor (aFGF), bFGF, NGF or glial cell–derived neurotrophic factor (GDNF) for local injection into the SCI site.^33^, ^34^, ^35^, ^36^, ^37^ The results showed that these single GF‐loaded injectable HP hydrogel achieved a certain improvement in promoting neuronal survival, axonal regeneration and functional recovery. However, nerve regeneration after SCI is a dynamic process that involves multiple GFs to modulate this process. Individual GF supplement is not sufficient to achieve successful axon regrowth across the lesion region. To overcome this difficulty, we encapsulated bFGF and NGF into HP hydrogel. Our results had revealed that this GFs‐HP hydrogel possessed robust effects on promoting the recovery of motor function, enhancing axon regeneration and reducing neurons apoptosis and glial scar formation. Furthermore, we also explored that the molecular mechanism of this GFs‐HP on repairing SCI was related to activate the MAPK/ERK and PI3K/Akt signalling pathways.

During the regeneration and development of the central nerve system, axon regeneration and synaptic connections are essential to achieve optimal functional recovery after SCI. Over the last few decades, numerous therapeutic strategies including transplantation of stem cells and/or biomaterials^38^, ^39^, ^40^ were aimed to improve neuroprotective and neuroregenerative capacity of damaged spinal cord, but positive clinical outcomes remained to be seen. The reasons that leaded to the failure of SCI repair should be attributed to the following reasons^41^, ^42^: (a) the weak growth capacity of damaged neurons; (b) the deterioration of regenerative microenvironment due to excessive inflammatory activation and myelin debris accumulation; and (c) glial scar, cystic cavity and inhibitory molecules formation. Therefore, how to ameliorate the intrinsic capacity of damaged neurons, how to create an optimal microenvironment for axonal regeneration and how to suppress the formation of glial scar and cystic cavities are critical issues for restoring SCI.

GFs belong to polypeptide therapeutic agents, which shows to exert multiple functions on neurogenesis, axonal growth, neuroprotection and revascularization. These attractive properties are considered for optimizing application of GFs in spinal cord repair. Based on the fact that different GFs exert distinctive activities and different neuronal subpopulations in the injured region of spinal cord need to apply different GFs, we consider that multiple GFs combinational treatment may be optimal as a feasible and effective therapeutic strategy for repairing SCI. In the present study, the reason of selecting NGF and bFGF together to treat SCI was considered as follows: (a) bFGF was regarded as a strong inducer for angiogenesis that was favour for supplying the nerve with oxygen and nutrients^43^, ^44^, ^45^, ^46^; (b) NGF promoted axonal regrowth and sprouting, remyelination and synaptic plasticity^47^; (c) their distinctive biological effects on angiogenesis and neurogenesis were able to enhance vessel‐nerve interaction, leading to synergistically facilitate functional recovery following SCI.^48^, ^49^, ^50^, ^51^ However, free application of both NGF and bFGF to repair SCI was hard to achieve an effective concentration at injured site, especially for the late stage of spinal cord repair. The reasons were including: both of them were biomacromolecules that possessed hard to penetrate the BSCB and easy degradation by proteolytic enzymes plus rapid diffusion in body fluids.^52^, ^53^, ^54^

In order to maintain their long‐term bioactivity, we employed the previous preparative heparin‐poloxamer (HP) hydrogel which consisted of Poloxamer 407 and heparin for delivering NGF and bFGF directly to the lesion area of damaged spinal cord. Poloxamer 407 possesses high biocompatibility that has been approved by US Food and Drug Administration (FDA) for biomedical application, such as vascular anastomosis.^25^ The ingredient of heparin has been demonstrated to combine multiple GFs with a high affinity. Thus, after embedding both GFs into HP, this GFs‐based delivery system not only released NGF and bFGF in a temporally and spatially controlled manner, but also protected them from proteolytic degradation. We have previously reported that the release characteristic of NGF and bFGF from this GFs‐HP exhibited an initial rapid phase during the first week, and a slow sustained release for prolonged time, with total release of bFGF and NGF from HP, was estimated to be 35% and 48%, respectively, over the 35‐day duration.^18^ Moreover, previous literatures had confirmed that HP hydrogel delivery of aFGF, bFGF, keratinocyte growth factor (KGF) or 17β‐estradiol exhibited remarkable therapeutic outcomes in various diseases, including SCI repair, wound healing or endometrial regeneration.

In the present study, we observed that the thermo‐sensitivity property and three‐dimensional network structure were not changed after incorporating NGF and bFGF into HP hydrogel (Figure 1). Moreover, compare with the orthotopic injection of free GFs or HP only, using this GFs‐HP hydrogel to repair chronic SCI contusion model provided more stronger and robust therapeutic efficacy and efficiency for inducing neuronal survival and axonal regrowth/plasticity, improving motor functional recovery, as well as reducing glial scar and cell apoptosis (Figures 2, 3, 4, 5, 6, 7, 8). These superior neuroprotective and neuroregenerative capacity indicate GFs‐HP is a safe and effective therapeutic drug for the treatment of chronic SCI. However, the related molecular mechanism of this superior effects is still unknown.

Accumulated evidence shows that MAPK/ERK and PI3K/Akt signalling pathways are particularly important for regulating neuronal survival and axonal regrowth under a wide variety of circumstance.^55^, ^56^, ^57^ Recent studies have implied the benefit effects of NGF on exerting neurogenesis and neuroprotection after acute SCI is related to bind Trk A receptor‐mediated activation of MAPK/ERK and PI3K/Akt axes.^47^, ^50^ Furthermore, the protective effects of bFGF on angiogenesis and functional recovery in contusive SCI rats were involved in bFGF interacting with its FGFR1 to activate MAPK/ERK and PI3K/Akt signalling cascades.^58^, ^59^ To verify whether GFs‐HP improves SCI structure and function was related to GFs‐HP constantly releasing NGF and bFGF to activate MAPK/ERK and PI3K/Akt signalling pathways via binding its corresponding receptors, we detected the protein expression of Trk A and FGFR1 receptors, and two signalling cascades‐related proteins using Western blotting. Results showed that the expression of Trk A and FGFR1, and the ratio of *P*‐Akt/Akt and *P*‐ERK/ERK were pretty low in SCI group. After administration of free GFs, this trend was significant reversed, but inferior to the GFs‐HP group (Figure 8). The reason might be explained that the sustained release of NGF and bFGF from GFs‐HP could invariably bind their corresponding receptors at a certain amount to persistently activate MAPK/ERK and PI3K/Akt signalling caspases, leading to continually repair SCI.

In conclusion, we describe here that a high affinity–binding hydrogel biomaterial with sustained release of bFGF/NGFF has the promising to serve as an effective therapeutic agent drug for improving SCI damage. This GFs‐HP has great biocompatibility and thermosensitive property, which is favourable for in situ administration. In the treatment of SCI, a single injection of this GFs‐HP manifests a great beneficial effect on the recovery of motor function. More importantly, GFs‐HP exhibited superior effects on neuroprotection and neuroregeneration, manifesting in promoting neuronal survival, enhancing axonal regeneration and plasticity, and attenuating neuronal apoptosis, as well as inhibiting glial scar former. Finally, we verify that the above therapeutic effects are probably achieved by activations of the MAPK/ERK and PI3K/Akt signalling pathways. Therefore, single injection of thermosensitive GFs‐HP hydrogel in situ may be a very promising strategy for the repair of SCI.

## CONFLICT OF INTEREST

The authors confirm that this article has no conflicts of interest.

## AUTHOR CONTRIBUTIONS

YJM and HXL designed the experiment and interpreted results. HXL performed experiments and drafted manuscript. LR made critical revision to manuscript. All authors have read and approved the final manuscript. YJM and XJ are co‐corresponding authors.

## Data Availability

The authors confirm that this article has no conflicts of interest.

## References

[1] Witiw CD , Fehlings MG . Acute spinal cord injury. J Spinal Disord Tech. 2015;28:202‐210. 26098670 10.1097/BSD.0000000000000287

[2] Chen Y , Tang Y , Vogel LC , et al. Causes of spinal cord injury. Top Spinal Cord Inj Rehabil. 2013;19:1‐8. 23678280 10.1310/sci1901-1 PMC3584795

[3] Orr MB , Gensel JC . Spinal cord injury scarring and inflammation: therapies targeting glial and inflammatory responses. Neurotherapeutics. 2018;15:541‐553. 29717413 10.1007/s13311-018-0631-6 PMC6095779

[4] Li R , Li D , Wu C , et al. Nerve growth factor activates autophagy in Schwann cells to enhance myelin debris clearance and to expedite nerve regeneration. Theranostics. 2020;10:1649‐1677. 32042328 10.7150/thno.40919 PMC6993217

[5] Li R , Li DH , Zhang HY , et al. Growth factors‐based therapeutic strategies and their underlying signaling mechanisms for peripheral nerve regeneration. Acta Pharmacol Sin. 2020 10.1038/s41401-019-0338-1 PMC7608263 32123299

[6] Matsumine H , Sasaki R , Tabata Y , et al. Facial nerve regeneration using basic fibroblast growth factor‐impregnated gelatin microspheres in a rat model. J Tissue Eng Regen Med. 2016;10:E559‐E567. 24737684 10.1002/term.1884

[7] Goldshmit Y , Frisca F , Pinto AR , et al. Fgf2 improves functional recovery‐decreasing gliosis and increasing radial glia and neural progenitor cells after spinal cord injury. Brain Behav. 2014;4:187‐200. 24683512 10.1002/brb3.172 PMC3967535

[8] Chen ZW , Wang HP , Yuan FM , et al. Releasing of herpes simplex virus carrying NGF in subarachnoid space promotes the functional repair in spinal cord injured rats. Curr Gene Ther. 2016;16:263‐270. 27903223 10.2174/1566523217666161121105717

[9] Zhao YZ , Jiang X , Xiao J , et al. Using NGF heparin‐poloxamer thermosensitive hydrogels to enhance the nerve regeneration for spinal cord injury. Acta Biomater. 2016;29:71‐80. 26472614 10.1016/j.actbio.2015.10.014 PMC7517710

[10] Zhu SP , Wang ZG , Zhao YZ , et al. Gelatin nanostructured lipid carriers incorporating nerve growth factor inhibit endoplasmic reticulum stress‐induced apoptosis and improve recovery in spinal cord injury. Mol Neurobiol. 2016;53:4375‐4386. 26232067 10.1007/s12035-015-9372-2

[11] Ferbert T , Child C , Graeser V , et al. Tracking spinal cord injury: differences in cytokine expression of IGF‐1, TGF‐ B1, and sCD95l can be measured in blood samples and correspond to neurological remission in a 12‐week follow‐up. J Neurotrauma. 2017;34:607‐614. 27533262 10.1089/neu.2015.4294

[12] Pacelli S , Acosta F , Chakravarti AR , et al. Nanodiamond‐based injectable hydrogel for sustained growth factor release: Preparation, characterization and in vitro analysis. Acta Biomater. 2017;58:479‐491. 28532899 10.1016/j.actbio.2017.05.026 PMC5560430

[13] Ohri SS , Hetman M , Whittemore SR . Restoring endoplasmic reticulum homeostasis improves functional recovery after spinal cord injury. Neurobiol Dis. 2013;58:29‐37. 23659896 10.1016/j.nbd.2013.04.021 PMC3748169

[14] Huang DW , McKerracher L , Braun PE , et al. A therapeutic vaccine approach to stimulate axon regeneration in the adult mammalian spinal cord. Neuron. 1999;24:639‐647. 10595515 10.1016/s0896-6273(00)81118-6

[15] Yoon JJ , Chung HJ , Lee HJ , et al. Heparin‐immobilized biodegradable scaffolds for local and sustained release of angiogenic growth factor. J Biomed Mater Res A. 2006;79:934‐942. 16941589 10.1002/jbm.a.30843

[16] Ishihara M , Obara K , Ishizuka T , et al. Controlled release of fibroblast growth factors and heparin from photocrosslinked chitosan hydrogels and subsequent effect on in vivo vascularization. J Biomed Mater Res A. 2003;64:551‐559. 12579570 10.1002/jbm.a.10427

[17] Perale G , Rossi F , Santoro M , et al. Multiple drug delivery hydrogel system for spinal cord injury repair strategies. J Control Release. 2012;159:271‐280. 22227024 10.1016/j.jconrel.2011.12.025

[18] Li R , Li Y , Wu Y , et al. Heparin‐poloxamer thermosensitive hydrogel loaded with bFGF and NGF enhances peripheral nerve regeneration in diabetic rats. Biomaterials. 2018;168:24‐37. 29609091 10.1016/j.biomaterials.2018.03.044 PMC5935004

[19] Liu Y , Lu JB , Chen Q , et al. Involvement of MAPK/ERK kinase‐ERK pathway in exogenous bFGF‐induced Egr‐1 binding activity enhancement in anoxia‐reoxygenation injured astrocytes. Neurosci Bull. 2007;23:221‐228. 17687397 10.1007/s12264-007-0033-y PMC5550585

[20] Ahmad I , Yue WY , Fernando A , et al. p75NTR is highly expressed in vestibular schwannomas and promotes cell survival by activating nuclear transcription factor κB. Glia. 2014;62:1699‐1712. 24976126 10.1002/glia.22709 PMC4150679

[21] Xu L , Tang YY , Ben XL , et al. Ginsenoside Rg1‐induced activation of astrocytes promotes functional recovery via the PI3K/Akt signaling pathway following spinal cord injury. Life Sci. 2020;252:117642. 32259600 10.1016/j.lfs.2020.117642

[22] Yoo MK , Cho KY , Song HH , et al. Release of ciprofloxacin from chondroitin 6‐sulfate‐graft‐poloxamer hydrogel in vitro for ophthalmic drug delivery. Drug Dev Ind Pharm. 2005;31:455‐463. 16093211 10.1080/03639040500214688

[23] Ye LB , Yu XC , Xia QH , et al. Regulation of Caveolin‐1 and Junction Proteins by bFGF Contributes to the Integrity of Blood‐Spinal Cord Barrier and Functional Recovery. Neurotherapeutics. 2016;13:844‐858. 27170156 10.1007/s13311-016-0437-3 PMC5147725

[24] Li R , Wu J , Lin Z , et al. Single injection of a novel nerve growth factor coacervate improves structural and functional regeneration after sciatic nerve injury in adult rats. Exp Neurol. 2017;288:1‐10. 27983992 10.1016/j.expneurol.2016.10.015

[25] Zhao YZ , Lv HF , Lu CT , et al. Evaluation of a novel thermosensitive heparin‐poloxamer hydrogel for improving vascular anastomosis quality and safety in a rabbit model. PLoS One. 2013;8:e73178. 24015296 10.1371/journal.pone.0073178 PMC3755001

[26] Dolbow DR , Credeur DP , Lemacks JL , et al. The effect of electrically induced cycling and nutritional counseling on cardiometabolic health in upper and lower motor neuron chronic spinal cord injury: dual case report. Int J Neurorehabil. 2019;6(1):336. 32149189 10.4172/2376-0281.1000336 PMC7059704

[27] Zhang F , Ying L , Jin J , et al. GAP43, a novel metastasis promoter in non‐small cell lung cancer. J Transl Med. 2018;16:310. 30419922 10.1186/s12967-018-1682-5 PMC6233536

[28] Gerin CG , Madueke IC , Perkins T , et al. Combination strategies for repair, plasticity, and regeneration using regulation of gene expression during the chronic phase after spinal cord injury. Synapse. 2011;65:1255‐1281. 21308793 10.1002/syn.20903

[29] Benowitz LI , Routtenberg A . GAP‐43: an intrinsic determinant of neuronal development and plasticity. Trends Neurosci. 1997;20:84‐91. 9023877 10.1016/s0166-2236(96)10072-2

[30] Silver J , Miller JH . Regeneration beyond the glial scar. Nat Rev Neurosci. 2004;5:146‐156. 14735117 10.1038/nrn1326

[31] Widenfalk J , Lundstromer K , Jubran M , et al. Neurotrophic factors and receptors in the immature and adult spinal cord after mechanical injury or kainic acid. J Neurosci. 2001;21:3457‐3475. 11331375 10.1523/JNEUROSCI.21-10-03457.2001 PMC6762497

[32] Guillemot F , Zimmer C . From cradle to grave: the multiple roles of fibroblast growth factors in neural development. Neuron. 2011;71:574‐588. 21867876 10.1016/j.neuron.2011.08.002

[33] Xu HL , Tian FR , Lu CT , et al. Thermo‐sensitive hydrogels combined with decellularised matrix deliver bFGF for the functional recovery of rats after a spinal cord injury. Sci Rep. 2016;6:38332. 27922061 10.1038/srep38332 PMC5138609

[34] Ko CC , Tu TH , Wu JC , et al. Acidic fibroblast growth factor in spinal cord injury. Neurospine. 2019;16:728‐738. 30653905 10.14245/ns.1836216.108 PMC6944993

[35] Wang Q , Zhang H , Xu H , et al. Novel multi‐drug delivery hydrogel using scar‐homing liposomes improves spinal cord injury repair. Theranostics. 2018;8:4429‐4446. 30214630 10.7150/thno.26717 PMC6134929

[36] Kojima A , Tator CH . Intrathecal administration of epidermal growth factor and fibroblast growth factor 2 promotes ependymal proliferation and functional recovery after spinal cord injury in adult rats. J Neurotrauma. 2002;19:223‐238. 11893024 10.1089/08977150252806974

[37] Chen G , Zhang Z , Wang S , et al. Combined treatment with FK506 and nerve growth factor for spinal cord injury in rats. Exp Ther Med. 2013;6:868‐872. 24137280 10.3892/etm.2013.1254 PMC3797285

[38] Yang L , Chueng SD , Li Y , et al. A biodegradable hybrid inorganic nanoscaffold for advanced stem cell therapy. Nat Commun. 2018;9:3147. 30089775 10.1038/s41467-018-05599-2 PMC6082841

[39] Papa S , Caron I , Erba E , et al. Early modulation of pro‐inflammatory microglia by minocycline loaded nanoparticles confers long lasting protection after spinal cord injury. Biomaterials. 2016;75:13‐24. 26474039 10.1016/j.biomaterials.2015.10.015

[40] Tsintou M , Dalamagkas K , Seifalian AM . Advances in regenerative therapies for spinal cord injury: a biomaterials approach. Neural Regen Res. 2015;10:726‐742. 26109946 10.4103/1673-5374.156966 PMC4468763

[41] Cui X , Chen L , Ren Y , et al. Genetic modification of mesenchymal stem cells in spinal cord injury repair strategies. Biosci Trends. 2013;7:202‐208. 24241170

[42] Jakovcevski I , Djogo N , Holters LS , et al. Transgenic overexpression of the cell adhesion molecule L1 in neurons facilitates recovery after mouse spinal cord injury. Neuroscience. 2013;252:1‐12. 23933311 10.1016/j.neuroscience.2013.07.067

[43] Lan L , Tian FR , ZhuGe DL , et al. Implantable porous gelatin microspheres sustained release of bFGF and improved its neuroprotective effect on rats after spinal cord injury. PLoS One. 2017;12:e0173814. 28291798 10.1371/journal.pone.0173814 PMC5349659

[44] Soto I , Marie B , Baro DJ , et al. FGF‐2 modulates expression and distribution of GAP‐43 in frog retinal ganglion cells after optic nerve injury. J Neurosci Res. 2003;73:507‐517. 12898535 10.1002/jnr.10673

[45] Rabchevsky AG , Fugaccia I , Turner AF , et al. Basic fibroblast growth factor (bFGF) enhances functional recovery following severe spinal cord injury to the rat. Exp Neurol. 2000;164:280‐291. 10915567 10.1006/exnr.2000.7399

[46] Zhang HY , Zhang X , Wang ZG , et al. Exogenous basic fibroblast growth factor inhibits ER stress‐induced apoptosis and improves recovery from spinal cord injury. CNS Neurosci Ther. 2013;19:20‐29. 23082997 10.1111/cns.12013 PMC6493620

[47] Zhang H , Wu F , Kong X , et al. Nerve growth factor improves functional recovery by inhibiting endoplasmic reticulum stress‐induced neuronal apoptosis in rats with spinal cord injury. J Transl Med. 2014;12:130. 24884850 10.1186/1479-5876-12-130 PMC4039547

[48] Hao W , Han J , Chu Y , et al. Collagen/Heparin bi‐affinity multilayer modified collagen scaffolds for controlled bFGF release to improve angiogenesis in vivo. Macromol Biosci. 2018;18:e1800086. 30160040 10.1002/mabi.201800086

[49] Pang Q , Zhang H , Chen Z , et al. Role of caveolin‐1/vascular endothelial growth factor pathway in basic fibroblast growth factor‐induced angiogenesis and neurogenesis after treadmill training following focal cerebral ischemia in rats. Brain Res. 2017;1663:9‐19. 28300551 10.1016/j.brainres.2017.03.012

[50] Salis MB , Graiani G , Desortes E , et al. Nerve growth factor supplementation reverses the impairment, induced by Type 1 diabetes, of hindlimb post‐ischaemic recovery in mice. Diabetologia. 2004;47:1055‐1063. 15184980 10.1007/s00125-004-1424-5

[51] Emanueli C , Salis MB , Pinna A , et al. Nerve growth factor promotes angiogenesis and arteriogenesis in ischemic hindlimbs. Circulation. 2002;106:2257‐2262. 12390957 10.1161/01.cir.0000033971.56802.c5

[52] Liu L , Lu B , Tu CQ , et al. Effect of basic fibroblast growth factor on the expression of glial fibrillary acidic protein after tractive spinal cord injury in rats. Chin J Traumatol. 2005;8:117‐120. 15769312

[53] Epstein SE , Fuchs S , Zhou YF , et al. Therapeutic interventions for enhancing collateral development by administration of growth factors: basic principles, early results and potential hazards. Cardiovasc Res. 2001;49:532‐542. 11166266 10.1016/s0008-6363(00)00217-0

[54] Kang CE , Tator CH , Shoichet MS . Poly(ethylene glycol) modification enhances penetration of fibroblast growth factor 2 to injured spinal cord tissue from an intrathecal delivery system. J Control Release. 2010;144:25‐31. 20114065 10.1016/j.jconrel.2010.01.029

[55] Mar FM , Simoes AR , Rodrigo IS , et al. Inhibitory injury signaling represses axon regeneration after dorsal root injury. Mol Neurobiol. 2016;53:4596‐4605. 26298667 10.1007/s12035-015-9397-6

[56] Glenn TD , Talbot WS . Signals regulating myelination in peripheral nerves and the Schwann cell response to injury. Curr Opin Neurobiol. 2013;23:1041‐1048. 23896313 10.1016/j.conb.2013.06.010 PMC3830599

[57] Calvo M , Zhu N , Grist J , et al. Following nerve injury neuregulin‐1 drives microglial proliferation and neuropathic pain via the MEK/ERK pathway. Glia. 2011;59:554‐568. 21319222 10.1002/glia.21124 PMC3222694

[58] Zhou Y , Wang Z , Li J , et al. Fibroblast growth factors in the management of spinal cord injury. J Cell Mol Med. 2018;22:25‐37. 29063730 10.1111/jcmm.13353 PMC5742738

[59] Zhang HY , Wang ZG , Wu FZ , et al. Regulation of autophagy and ubiquitinated protein accumulation by bFGF promotes functional recovery and neural protection in a rat model of spinal cord injury. Mol Neurobiol. 2013;48:452‐464. 23516099 10.1007/s12035-013-8432-8

